# Low Expression of miR-424-3p is Highly Correlated with Clinical Failure in Prostate Cancer

**DOI:** 10.1038/s41598-019-47234-0

**Published:** 2019-07-23

**Authors:** E. Richardsen, S. Andersen, S. Al-Saad, M. Rakaee, Y. Nordby, M. I. Pedersen, N. Ness, L. M. Ingebriktsen, A. Fassina, K. A. Taskén, I. G. Mills, T. Donnem, R. M. Bremnes, L. T. Busund

**Affiliations:** 1 Translational Cancer Research Group, Institute of Medical Biology, UiT The Arctic University of Norway, Tromso, Norway; 2 Translational Cancer Research Group, Institute of Clinical Medicine, UiT The Arctic University of Norway, Tromso, Norway; 3 Department of Clinical Pathology, University Hospital of North Norway, Tromso, Norway; 4 Department of Oncology, University Hospital of North Norway, Tromso, Norway; 5 Department of Urology, University Hospital of North Norway, Tromso, Norway; 6 Department of Medicine, University of Padua, 35121 Padova, Italy; 7 Institute of Cancer Research, Oslo University Hospital, Oslo, Norway; 8 Centre for Cancer Research and Cell Biology, Queen’s University of Belfast, Belfast, UK; 9 Institute of Clinical Medicine, University of Oslo, Oslo, Norway; 10 Nuffield Department of Surgical Sciences, University of Oxford, Oxford, UK

**Keywords:** Urological cancer, Cancer

## Abstract

Prostate cancer (PC) is a highly heterogenous disease and one of the leading causes of mortality in developed countries. Recently, studies have shown that expression of immune checkpoint proteins are directly or indirectly repressed by microRNAs (miRs) in many types of cancers. The great advantages of using miRs based therapy is the capacity of these short transcripts to target multiple molecules for the same- or different pathways with synergistic immune inhibition effects. miR-424 has previously been described as a biomarker of poor prognosis in different types of cancers. miR-424 is also found to target both the CTLA-4/CD80- and PD-1/PD-L1 axis. In the present study, the clinical significance of miR-424-3p expression in PC tissue was evaluated. Naïve radical prostatectomy specimens from 535 patients was used for tissue microarray construction. *In situ* hybridization was used to evaluate the expression of miR-424-3p and immunohistochemistry was used for CTLA-4 protein detection. In univariate- and multivariate analyses, low expression of miR-424-3p was significant associated with clinical failure-free survival, (p = 0.004) and p = 0.018 (HR:0.44, CI95% 0.22–0.87). Low expression of miR-424-3p also associated strongly with aggressive phenotype of PC. This highlight the importance of miR-424-3p as potential target for therapeutic treatment in prostate cancer.

## Introduction

It has become increasingly evident that the immune system represents an important option for the development of anticancer treatment. Most of the anti-cancer research has focused on immunotherapy, which aims to enhance antitumor immunity by blocking immune check-points (ICPs)^1^. In 2010 the immune therapy cancer vaccine sipuleucel-T was approved by the US Food and Drug Administration (FDA) for men with metastatic castration resistant PC (mCRPC)^2^ and the ongoing early phase trials of programmed cell death protein-1 (PD-1) inhibitors have reported promising results in prostate cancer (PC)^2–4^. Latest, results from a phase 1 study with pembrolizumab treatment in programmed cell death ligand -1 (PD-L1) positive PC, showed that the median response treatment time was 13.5 months, progression-free survival and overall survival were 3.5 and 7.9 months, respectively^4^. However, there have been several notably immunotherapy failures in PC and recent studies has demonstrated that the tumor microenvironment (TME) might be the reason for this because the TME might be predisposed towards immunosuppression^5–8^.

mRNAs (miRs) are a class of non-coding small RNA molecules and are found to be key regulators of gene expression which regulate metabolic- and cellular pathways for controlling cell proliferation, differentiation and survival. Recently, studies of the relationship between miRs, ICPs and how miRs modulate the immunity via ICPs, have received increasing attention^9–12^. ICP molecules can up- or downregulate important signals that can lead to modulate different immune actions^9^. In a cancerous tissue, the tumor cells can dysregulated the expression of ICPs on immune cells in order to suppress the antitumor immune response which can lead to immune resistance, consequently aiding in progression of various cancers^13,14^. In a recent study by Xu *et al*. in ovarian cancer cell lines and in ovarian cancer tissues, they found that the expression of miR-424 was negatively correlated with the expression level of cytotoxic T-lymphocyte associated protein 4 (CTLA-4), PD-L1 and CD80. Further, they observed higher activity of T cells and reversion on chemoresistancy following by restoration of miR-424^12^, which increased the progression-free survival time. If miR-424 biologically mimics the activity to the expression of ICPs as a regulator of both directly and the indirectly control mechanisms this will be important knowledge. MiRs which targets multiple checkpoint molecules could be unique therapeutic targets because they mimic the blockade with multiple immune checkpoint inhibitors which found to be superior to single antibody therapy^12,14–16^. CTLA-4 is a protein receptor that downregulates immune responses and appears on the surface of T cells which is activated on by contact with antigen and acts to inhibit lymphocytes response^12^. Moreover, in a tumor progression situation the miR-424 can directly block the binding of PD1/PDL1 and CD80/CTLA-4 action and thereby induce tumor suppression^17^.

Specifically, based on published data showing that miR-424 is inversely correlated with CTLA-4 we wanted to investigate the prognostic significance of miR-424-3p and CTLA-4 in untreated radical prostatectomy specimens (no = 535) with extensive follow-up. Further we analysed their potential role in a large PC cohort and correlation to clinicopathological variables and previous published biomarkers, PD-L1/PD-1 and subsets of T cells was assessed.

## Materials and Methods

### Patients

Radical prostatectomy specimens of 671 patients were retrospectively collected from two different Health care regions in Norway: Northern Norway Regional Health Authority and Central Norway Regional Health Authority. The inclusion time period was from 1995 to 2005. All patients were diagnosed with adenocarcinoma. Of these, 131 patients were excluded due to (1) neoadjuvant therapy prior surgery, (2) other types of cancer 5 years before PC diagnosis, (3) missing clinical follow-up data, and (4) inadequate paraffin-embedded tissue blocks, leaving 535 prostatectomy specimens available for investigation. None of the patients had received pre-operative hormonal therapy. From the patients’ records the following variables were registered: demographical data, age at surgery, surgical procedures, preoperative serum PSA level, postoperative therapy (radio-, hormonal- and/or chemotherapy), and outcome data until the last follow-up date December 31, 2015. Median follow-up was 12.4 years (range 1.5–20 years). Biochemical failure-free survival (BFFS) was calculated as time from surgery to last follow-up (FU) date, or date with postoperative PSA ≥ 0.4 ng/ml or intervention with adjuvant therapy. Clinical failure-free survival (CFFS) was defined as clinically palpable tumor recurrence or metastasis verified by radiology. Prostate cancer specific death free survival (PCDFS) was defined as death caused by PC stated in the patient’s journal. Detailed information regarding the cohort are previously published^18^.

### Tissues and tissue microarray construction (TMAs)

Tumor tissue consisting of formalin-fixed paraffin-embedded tissue blocks from prostatectomy specimens were used. One experienced uro-pathologists (ER) re-evaluated the tumors and classified them according to the recent updated Gleason grading system^19,20^ and staged according to the new guidelines^21^. For high-throughput molecular investigation we used TMA and twelve TMA blocks were constructed. From paraffin-embedded blocks the uro-pathologist (ER) identified and marked the most representative areas of viable neoplastic epithelial cells and adjacent tumor stromal areas. The detailed methodology has been reported previously^21^. Briefly, we used a 0.6 mm-diameter stylet, and the study specimens were routinely sampled with duplicate cores samples from different areas of neoplastic tissue and tumor stroma (totally 4 cores for each patient).

### *In situ* hybridization (ISH)

Chromogenic ISH was performed on Ventana Discovery Ultra instrument (Ventana Medical Inc, Arizona, USA). Buffers and detection reagents were purchased from Roche (Basel, Switzerland). miRCURY LNA detection probe and controls was from Exiqon (Vedbaek, Denmark). Exiqon validated the LNA^TM^ oligonucleotides by Capillary Electrophoresis (EC) and High-Performance Liquid Chromotography (HPLC) and confirmed the identity of compounds using Mass Spectrometry.

### Optimization of method and probes

**Figure 1:**
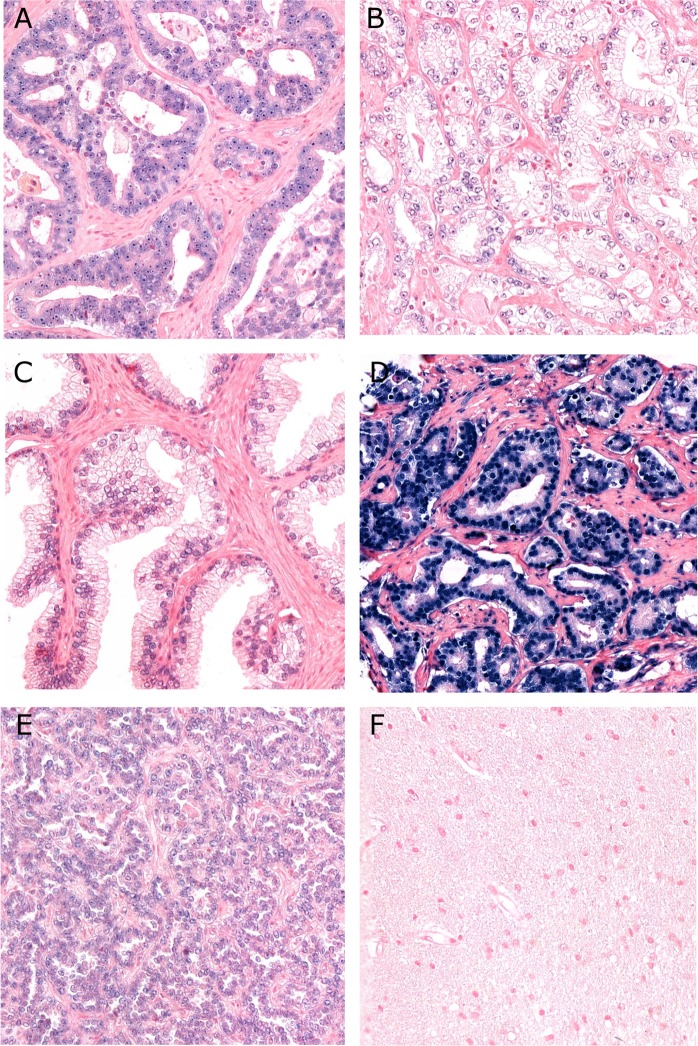
Panel of miR-424-2p *in situ* hybridization staining. (**A**) Strong nuclear staining of miR-424-3p, Gleason grade group 4 (4 + 4); (**B**) Weak nuclear staining of miR-424-3p, Gleason grade group 1 (3 + 3); (**C**) Normal prostatic tissue with some nuclear staining of miR-424-3p; (**D**) U6 control staining; (**E**) Positive tissue control: papillary renal cell carcinoma; (**F**) Negative tissue control: normal human brain tissue. (magnification 20x).

To prevent RNA degradation, we used RNAse-free water in buffers during the sectioning. Probe concentrations and unmasking pretreatments was tested on one TMA multi-organ block to optimize the detection method. Hybridization temperatures for each probe and controls was tested and a set with RNA melting temperature as a guideline. The sensitivity level of ISH method was ensured by the use of U6 snRNA control probe at 0.5 nM concentration. Nuclear signal at concentrations between 0.1–2.0 nM for U6 was considered to have the best sensitivity. U6 also indicated low degree of RNA degradation by visualizing strong nuclear staining by light microscope. Scramble miR negative control probe gave no unspecific positive staining in prostate TMA cores. For validation purpose, miR-424-3p staining expression was investigated on one multi-organ control TMA block which included both normal and tumor tissues (Fig. 1).

### ISH procedure

The ISH protocol steps are documented in our previous study^22^. In detail for miR-424-3p probing, TMA blocks were sectioned with 4 µm thickness and incubated at 60 °C. Liquid Coverslip oil (Roche, 5264839001) was used to protect sections from drying and also securing proper incubation of regents across slide. Deparaffinization was performed by EZ Prep buffer (Roche, 5279755001) at 68 °C (3 × 12 min). Target unmasking to disengage cross linking effect of formalin fixation was done at 95 °C with CC2 buffer (Roche, 6414575001) for 40 minutes to allow the DIG labeled LNA^TM^ probes to hybridize to the patient microRNA sequence. After target unmasking, the sections were rinsed with Reaction Buffer (Roche, 5353955001) and then RiboWash, SSPE buffer (Roche, 5266262001). Probes were diluted in a 1:1 solution of Exiqon microRNA ISH buffer (9000) and Elix RNAse free water to their final concentration. 10 nM miR-424-3p (Exicon, 611050-360), 10 nM Scramble miR (Exiqon, 99004-15) negative control probe and 0.5 nM U6 (Exiqon, U6, 99002-15) positive control probe was used in this study.

Denaturation of the LNA-probes was achieved at 8 min at 90 °C to get optimal hybridization conditions. Hybridization of the LNA-probes miR-424-3p, was performed in the instrumnet for 60 min at 55 °C for miR-424-3p, 57 °C for scramble miR and 55 °C for U6. Stringent washes was done 2 × 8 min with 2.0X RiboWash, SSPE buffer with same temperatures as used under hybridization for each probe. Blocking against unspecific bindings then followed with blocking solution (Roche, 5268869001) for 16 min. at 37 °C. Alkaline phosphatase (AP)-conjugated anti DIG (Anti-DIG-AP Multimer, Roche, 07256302001) was incubated for 20 min at 37 °C for immunologic detection. After rinsing substrate enzymatic reactions was carried out with NBT/BCIP (CromoMap Blue kit, Roche, 526661001) 60 min. at 37 °C to give a blue precipitate to detect the microRNA. Sections were again rinsed and counterstained 4 min with Red Stain II (Roche, 5272017001). After manual washing in tap water, dehydration was done by increasing gradients of ethanol solutions to Xylene. In the end, sections were mounted with Histokitt mounting medium.

### Immunohistochemistry (IHC)

Discovery-Ultra was used for IHC analysis of CTLA-4 expression. Mouse monoclonal CTLA-4 (CD-152) antibody, clone 14D3, (eBioscience, cat#14-1529-80), was used in this study. Antibody concentrations and unmasking pretreatments was tested using both TMA and whole tissue slides. Positive and negative tissue controls, and negative subclass isotype-matched control antibody (Biolegend, cat#400203) was included.

Deparaffinization was performed in EZ Prep buffer (Roche, 5279755001) at 68 °C (3 × 12 min). Target unmasking to disengage cross linking effect of formalin fixation was done at 95 °C with CC1 buffer (Roche, 6414575001) for 24 minutes. Endogenous peroxidase was blocked for 8 minutes by Discovery inhibitor CM (Roche, 05266645001). Mouse monoclonal CTLA-4 antibody (1:100 dilution) was incubated for 32 minutes at 36 °C. Secondary multimer antibody OmniMap anti-Ms HRP (Roche, 5269652001) followed as immunologic detection for 16 minutes and substrate reaction was done with Discovery ChromoMap DAB detection kit (Roche, 526645001). All sections were counterstained for 16 minutes with hematoxylin (Roche, 5266726001) and post counterstained for 4 minutes with bluing Reagent, (Roche, 5266769001).

### Antibody validation

Validation and details of western blotting have been presented in our previous lung cancer study^23^. Briefly, CTLA-4 protein expression was detected using the CD-152 mouse monoclonal antibody (1:100 dilution, eBioscience, Cat#14-1529-80). To validate the specificity of the antibody on Western blot, HEK293 transfectant cell lysates with and without CTLA-4 expression were analysed as described. Actin was used as loading control on the Western Blot by using an anti-actin antibody (1:10000 dilution, Sigma-Aldrich, Cat#A2066).

The specificity of the antibody in IHC analyses was verified by staining multi-organ TMA as negative and positive controls. We included TMA tissue controls (positive: papillary renal cell carcinoma and negative: normal brain) and negative method controls in each staining run. Each TMA slide, from the main cohort, additionally contained normal prostate epithelium and stroma that acted as internal controls. As negative staining controls—the primary antibody was omitted to observe whether the secondary antibody or some other elements in the system are reacting with a component of the tissue.

**Figure 2:**
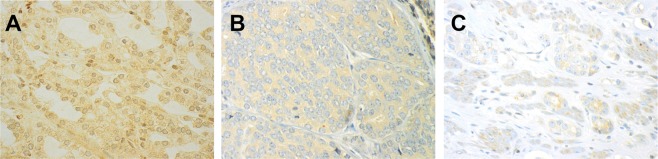
Panel of CTLA-4 immunohistochemical staining. (**A**) Strong nuclear staining expression of CTLA-4, Gleason grade group 1 (3 + 3); (**B**) Moderate staining expression, Gleason grade group 4 (4 + 4); (**C**) Weak staining expression, Gleason grade group 5 (4 + 5). (magnification 20x).

Scoring and expression of miR-424-3p and CTLA-4. The samples were anonymized and independently scored by two observers (miR-424-3p: ER, SAS, CTLA-4: ER, MR). During the assessment of a given score, the two observers were blinded to each other’s findings, clinicopathological variables, and outcomes. In case of disagreement (scoring difference >1) the core was re-examined and consensus was reached. miR-424-3p was expressed in the nuclei of tumor cells. The staining intensity was all over strong (Fig. 1A,B). We also observed some staining in normal cells, but the staining intensity was mostly weak (Fig. 1C) For miR-424-3p, the percentage of positively blue-coloured tumor epithelial cell nuclei was counted, and subsequently given a score using the following system: 0 = 0%, 1 = 1–2.5%, 2 = 2.6–4%, 3 ≥5%. The scoring for miR-424-3p was then dichotomized to low vs high score, defined as mean <3.62 (low score) and ≥3.62 (high score). CTLA-4 was investigated in epithelial tumor cells (TE), adjacent tumor stromal areas (TS) and TE + TS as one compartment. The staining of CTLA-4 positive cells was overall weak, relatively homogenous and granular in tumor tissue, both in the TE and TS (Fig. 2). In most cases the staining was located in the cytoplasm. Because of homogenous staining the percentage (density) did not add valuable information to the score and was therefore not included. Intensity of CTLA-4 staining was scored as no signal = 0, weak = 1, moderate = 2 and strong = 3 (Fig. 2A–C). For CTLA-4 (TE + TS) low score was defined as <2.29 (mean), and a high score as ≥2.29.

Cut-off values for dichotomization were chosen according to a minimal p-value (p < 0.005) approach securing statistically significant number in each group. For both, the optimal threshold value was defined as mean.

### Statistical methods

The SPSS software, version 24 (IBM, SPSS Inc., Chicago, IL, USA) was used for all analyses. Inter-observer reliability between the pathologists (ER/SAS, ER/MR) was tested by use of a two-way random effect model (absolute agreement). Spearman correlation test was used to examine the association between miR-424-3p, CTLA-4, clinicopathological variables, subsets of T cells^24^, PD-1 and PD-L1^25^. In univariate analysis of survival according to miR-424-3p and CTLA-4, the Kaplan-Meier method were applied, and statistically significant differences between survival curves were analyzed by the log-rank test. For multivariate analyses the Cox-regression analysis was used with a proportional model, testing the probability for stepwise entry at 0.05 and stepwise removal was set at 0.05 and 0.10, respectively. P-values < 0.05 was considered statistically significant.

### Ethics

This study was approved by the Regional Committees for Medical Health Research Ethics (REK Nord), Ref. no: 2009/1393. A mandatory re-approval was conducted in 2016 and 2019. REK Nord considered written consent is not necessarily due to retrospective nature of this study, and as the majority of the material is more than 10 years old and many of the patients deceased. The Norwegian Centre for Research Data (NSD) approved the assembly of the database. Prior the study, all included was made anonymous and given a trial number which were used during the study. The reporting of clinicopathological information, survival data and biomarker expression status was conducted in accordance with the REMARK guidelines^25^.

## Results

### Patient characteristics

Table 1 provide the clinical- and histopathological data for all 535 patients. Median age at surgery was 62 years (range 47–76). The surgical procedures were retropubic in 81% (n = 435) and perineal in 19% (n = 100). Gleason grade group ranged from 1 to 5; 1 (≤6), 2 (3 + 4), 3 (4 + 3), 4 (4 + 4) and 5 (≥8). Tumor stage included T2a to T3b. PSA was 8.8 (median range 0.7–104). At last follow-up, 200 (37%) had BF, 56 (11%) had CF and 18 (3.4%) had died of PC.

**Table 1:** Prognostic clinicopathologic variables as predictors of BF, CF and DSS in 535 prostate cancer patients (univariate analysis; log-rank test).

Characteristics	Patients n (%)	BFFS (n = 200) 5-year EFS (%)	*P*	CFFS (n = 56) 10-year EFS (%)	*P*	DSS (n = 18) 10-year EFS (%)	*P*
**Age**			0.237		**0.038**		0.404
**<65 year**	357 (37)	77		94		98	
≥65 year	178 (33)	70		91		98	
**Preop. PSA**			**<0.001**		**0.029**		**0.003**
PSA < 10	308 (57)	81		95		99	
PSA > 10	221 (42)	68		89		97	
**pT-stage**			**<0.001**		**<0.001**		**0.001**
pT2	374 (70)	83		97		99	
pT3a	114 (21)	61		87		98	
pT3b	47 (9)	43		74		91	
**pN-stage**			**<0.001**		**<0.001**		**<0.001**
NX	264 (49)	79		96		99	
N0	268 (50)	72		90		97	
N1	3 (1)	0		33		67	
**PNI**			**<0.001**		**<0.001**		**<0.001**
No	401 (75)	80		96		99	
Yes	134 (25)	60		83		95	
**Tumor size**			**<0.001**		**0.002**		0.085
<20 mm	250 (47)	83		96		99	
≥20mm	285 (53)	68		90		97	
**PSM**			**0.049**		0.198		0.843
No	249 (47)	81		90		98	
Yes	286 (53)	69		96		98	
**Apical PSM**			0.063		0.427		0.128
No	381 (71)	82		96		99	
Yes	154 (29)	57		85		96	
**Non-apical PSM**			**<0.001**		**<0.001**		**0.022**
No	381 (71)	82		96		99	
Yes	154 (29)	57		85		96	
**LVI**			**<0.001**		**<0.001**		**<0.001**
No	492 (92)	77		95		99	
Yes	43 (8)	47		70		90	
**Surgical proc**.			0.466		0.308		0.965
Retropubic	435 (81)	77		92		98	
Perineal	100 (19)	68		95		99	
**Gleason grade Group**			**<0.001**		**<0.001**		**<0.001**
1 (≤ 6)	183 (34)	83		98		99	
2 (3 + 4)	219 (41)	77		94		99	
3 (4 + 3)	81 (15)	70		90		96	
4 (4 + 4)	17 (3)	58		86		94	
5 (>8)	35 (7)	36		65		91	

Abbreviations: BFFS = biochemical failure-free survival; CFFS = clinical failure-free survival; DSS = disease specific survival; EFS = event free survival; LVI = lympho-vascular infiltration; PNI = perineural infiltration; preop = preoperative; PSA = prostate specific antigen; PSM = positive surgical margin; Surgical proc. = surgical procedure.

### Correlations

ICC between the scores ER/SAS was 0.97 (CI: 0.91–0.98) and between ER/MR 0.093 (CI: 0.89–0.98). We correlated the expression of miR-424-3p to all clinicopathological variables due to their possible association to prognosis (Table 1). We found significant correlations between mir-424-3p and the following clinicopathological variables: high Gleason grade group (*r* = 0.12, p = 0.014), Gleason grade group ≥8 (*r* = 0.11, p = 0.024), large tumor size (>20 mm, *r* = 0.13, p = 0.013), perineural infiltration (*r* = 0.11, p = 0.030) and vascular infiltration (*r* = 0.12, p = 0.014). CTLA-4 did not correlate to any clinicopathological variables. We also made correlations between miR-424-3p, CTLA-4 and our previously published ICP (PD-1/PD-L1)^25^ and subset of T cells (CD3, CD4, CD8 and CD20)^24^. miR-424-3p correlated significantly to CTLA-4, *r* = 0.10, p < 0.001, and to PD-L1 (TE compartment, cut-off: mean value = 0), *r* = 0.11, p = 0.040. CTLA-4 correlated to PD-1 (TE compartment, cut-off: mean value = 1.0), *r* = 0.10, p = 0.054, and PD-1 (TS compartment, cut-off: mean value = 0.54), *r* = 0.16, p = 0.002). Correlation to subsets of T cells, miR-424-3p did not correlate to the prevalence of any T- cells. CTLA-4 correlated to CD3+ T cells (*r* = −0.11, p = 0.028), CD4+ T cells (*r* = −0.12, p = 0.009). Detailed information regarding PD-1/PD-L1 and the subset of T-cells, IHC, validation, scoring methods, staining protocol, staining localization and statistical analyses are described in detail previously^22,24^.

### Univariate analyses

**Figure 3:**
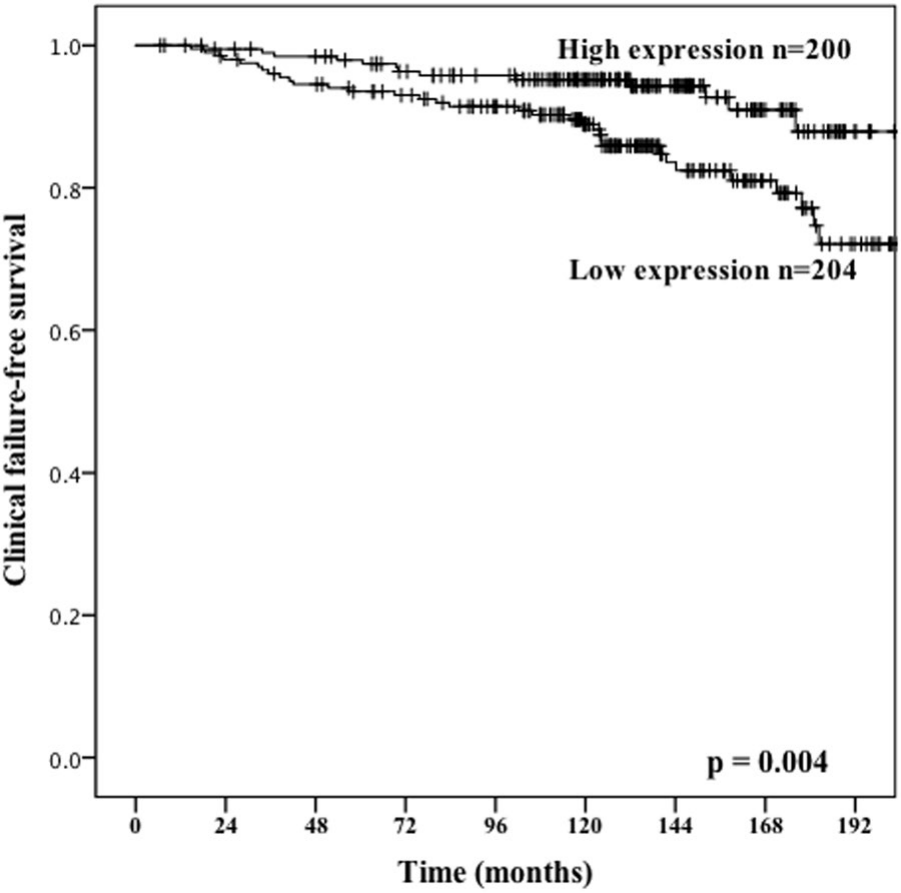
Association with prostate cancer outcome and miR-424-3p. Kaplan-Meier survival curves displaying clinical failure-free survival (CFFS) dichotomized into high vs low expression of miR-424-3p. Low score was defined as <3.62 and high score to ≥3.62.

Results from the univariate analysis are presented in Table 1 and Fig. 3. The following clinicopathological variables were significant prognostic factors for BF; pre-operative PSA (p < 0.001), pT stage (p < 0.001), pN stage (p < 0.001), perineural infiltration (p < 0.001), tumor size (p < 0.001), non-apical PSM (p < 0.001), vascular infiltration (p < 0.001), and Gleason grade group (p < 0.001). miR-424-3p and CTLA-4 (TE, TS, TE + TS) was not significant for BF. For CF; age (p = 0.038), PSA (p = 0.029), pT stage (p < 0.001, pN stage (p < 0.001), tumor size (p = 0.002), non-apical PSM (p < 0.001), vascular infiltration (p < 0.001), Gleason score (p < 0.001) and miR-424-3p (p = 0.004). CTLA-4 was not significant (TE + TS, p = 0.093). For PCD; preoperative PSA (p = 0.003), pT stage (p < 0.001), pN stage (p < 0.001) perineural infiltration (p < 0.001), non-apical positive surgical margin (p = 0.022) and Gleason score (p < 0.001).

### Multivariate analyses

Results from the multivariate model is present in Table 2. The following clinicopathological variables were significant with poor BFFS: perineural infiltration, pT stage, pT3b, Gleason grade group, Gleason grade group ≥4, and both, apical- and non-apical PSM. Low expression of miR-424-3p (HR: 0.44, 95% CI 0.22–0.87, p = 0.018) was associated with aggressive disease and poor CFFS together with aggressive feature of PC.

**Table 2:** miR-424-3p expression and significant clinicopathological variables (Multivariate model, Cox regression, backward conditional).

Characteristic	No	BF (200 events)			CF (56 events)			PCD (18 events)		
		HR	95% CI	*P*	HR	95% CI	*P*	HR	95% CI	*P*
**Age**				NS			NS			NS
≤65 years	264									
>65 years	135									
**pT-stage**				**0.035**			NS			NS
pT2	275	1								
pT3a	85	1.42	(0.69–2.86)	0.343						
pT3b	39	0.52	(0.32–0.83)	**0.006**						
**Preoperative PSA**				NS			NS			**0.023**
PSA <10	224							1		
PSA >10	175							0.21	(0.06–0.81)	
**Gleason grade group**				**0.016**			**0.001**			NS
1 (3+3)	126	1			1					
2 (3+4)	231	2.13	(0.47–9.70)	0.329	0.19	(0.03–1.19)	0.076			
3 (4+3)	115	2.90	(0.66–12.82)	0.161	0.56	(0.12–2.62)	0.465			
4 (4+4)	24	4.25	(0.82–22.13)	0.085	1.27	(0.20–8.18)	0.804			
5 (≥9)	3	4.57	(1.00–20.80)	**0.050**	2.32	(0.49–11.03)	0.290			
**Tumor size**				NS			NS			NS
0–20 mm	169									
>20 mm	230									
**PNI**				**0.008**			NS			NS
No	298	1								
Yes	101	0.62	(0.44–0.83)							
**Non-apical PSM**				**0.003**			NS			NS
No	284	1								
Yes	115	0.58	(0.41–0.83)							
**Apical PSM**				**0.02**			NS			NS
No	247	1								
Yes	152	1.52	(1.07–2.15)							
**Vascular infiltration**				NS			**0.008**			NS
No	365				1					
Yes	34				0.39	(0.19–0.79)				
**miR-424-3p**				NS			**0.018**			NS
Low	200				1					
High	204				0.44	(0.22–0.87)				

Abbreviations: BF = biochemical failure; CF = clinical failure; PCD = prostate cancer death; PSA = prostate specific antigen; PNI = perineural infiltration, PSM = positive surgical margin; NS = not significant.

## Discussion

In this study, we identified that low expression of the miR-424-3p was significant predictor for PC aggressiveness and outcome and was associated with aggressive features in PC; high Gleason grade group, large tumor size, perineural- and vascular infiltration. We also found that low expression of miR-424-3p was associated with worse clinical failure-free survival. Furthermore, a positive correlation between miR-424-3p and CTLA-4 was observed, indicating a functional pathway between miR-424-3p and this ICP molecule. Further, CTLA-4 was significantly correlated with CD3+ and CD4+ T cells. To the best of our knowledge this is the first large-scale study investigating the miR-424-3p and CTLA-4 pathway in untreated human PC specimens. The strength of this study is the large number of patients, the long extensive clinical follow-up data and the *in-situ* examination of in both tumor epithelial cells and adjacent tumor stromal area. We did not, however, have paired normal tissue controls and we did not perform cell line studies for validation purpose, which are limitations to this study.

The clinical success of several anti-PD-1 and anti-CTLA-4 treatment in various malignancies has boosted the research of immune checkpoint pathways^26–29^. In PC, however these treatment modalities have been relatively disappointing^6–8,30^, and progression to a chemo-resistant, androgen-independent state is the norm. Only a small number of subset of patients respond to current available immunotherapies^31^, which may indicate that prostate cancer is different from immunogenic cancers as melanoma and lung^25–29,32^.

**Figure 4:**
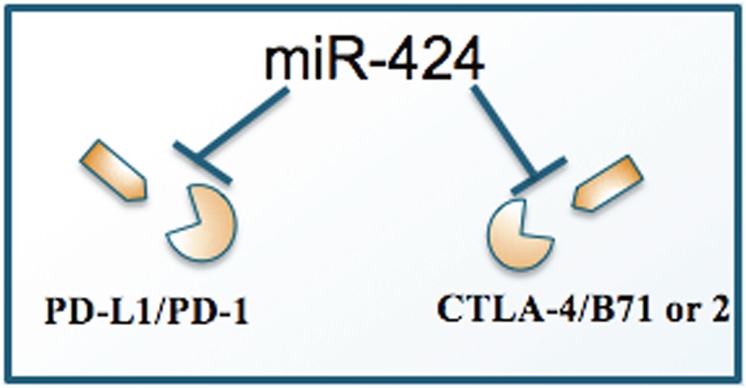
Schematic presentation of the interaction between miR-424 and the immune checkpoint proteins, PD1/PD-L1 and CTLA-4.

Recent studies have shown that PC tumor microenvironment (TME) may play an important role in explaining treatment failure^5–8^. The major explanation so far is that the TME is largely heterogeneous and that PC is not a hypermutated disease as other urological cancers^16^ and that absence of successfully treatment modalities as blocking the axis of PD-1/PD-L1 and CTLA-4/CD80 expression is due to several alterations in these ICP molecules^9,12,13^. The role of miRs as regulators of ICP molecules have been intensively investigated and discussed in several studies^9–12^ and a large body of evidence has indicated that several miRs play an important role in regulation of the host immune response and ICP molecules^10,11,14,16^ and attention has been drawn to miRs that directly or indirectly controls expression of more than one ICP molecule (Fig. 4), and vice versa. Although the role of miR-424 in different type of cancers have been reported, these studies are conflicting, both tumor-suppressing and tumor promoting functions have been proposed^31,33–35^. Noteworthy, the understanding of miR-424 comes derives primarily from mechanistic studies on cell lines and animal models, little from studies on human tissues, or if, on human tissue the number included is limited. Additionally, many of the functionally studies were limited to the use of cell lines in which miR-424 were overexpressed. This may also account for the discrepancies. miR-424 belongs to the miR-16 family and members of this family have previously shown to be fundamental targets for cell cycle arrests and apoptosis^15^. Furthermore, miR-424 is found to be predicted to target many miRs, epidermal growth factor receptor (EGFR), Twist and DNA damage-repair genes^17^. They evaluated the finding from *in vitro s*tudies by IHC of clinical samples and found that miR-424 upregulation was negatively correlated with EGFR expression^17^. The same group also reported that a downregulation of miR-424 was associated with adverse prognostic factors such as advanced clinical stages, nodal metastasis, and high pathological grades^17^. Recently, Zhang *et al*. reported a functional role of miR-424/Akt3/E2F3 axis in hepatocellular carcinoma development, suggesting that miR-424 could be a potential indicator of prognosis^36^. In PC there is only a limited number of studies and they all has limitation mentioned above. In a study by Dallavalle *et al*. miR-424 expression was evaluated in both normal- (no = 21) and PC tissues (no = 48) and cell lines^37^, miR-424 was found to be highly expressed in metastatic subclones of DU145 cells and promoted the EMT and metastatic phenotype, which is consistent with Banyard *et al*. study^38^. In the latter, they found that stable MiR-424 expression reduced epithelial-mesenchymal progression. Review- and met-analyses miR-screening studies on different PC cell lines and miR-424 did not report any significant findings^39–41^. Palladine *et al*. linked miR-424 to the innate immune system by activate monocytes to differentiate and activate^42^ however, here we failed to correlate MiR-424-3p to any monocytes.

Based on the above-mentioned mechanistic studies and a recently published study by Xu *et al*.^12^ miR-424 is appearing as one novel and important biomarker that has the potential to predict tumor progression and treatment target option. In the study by Xu *et al*. they found that low levels of miR-424(322) was associated with chemoresistance and that miR-424(322) was inversely correlated with the PD-1/PD-L1 and CTLA-4/CD80 pathways, by inhibiting PD-L1 and CD80^12^.

In general, the lack of data regarding the targets of miR-424 hampers a full understanding of the biological functions of aberrant miR-424.

In PC, the knowledge about of miR-424 is very limited. Our study is the first large-scale study investigating the miR-424-3p and CTLA-4 pathway in human prostatectomy specimens (no = 535), we also abled to investigate the correlation to PD1, PD-L1 and subset of T cells. Herein, we found that a low expression of miR-424-3p was significantly correlated to aggressive PC phenotype, worse clinical outcome and reduced time for clinical failure, which is in line with previous *in vitro* and *in vivo* studies^36,38,42–46^. We also identified a correlation between miR-424-3p, CTLA-4 and PD-L1, which is in accordance with one previous report^12^.

What is well known is that PC tissue is marked by large T cell inflammatory infiltrates within TE and TME participating in host defence mechanisms against tumor cells^40,41^ and that PD-1/PD-L1 pathway has a crucial role in the regulating of T cell activation during inflammatory processes^33^, in contrast to CTLA-4, which inhibits T cell activityduring the T cell priming phase^41,42^. Here, we found that miR-424-3p correlated significantly to CTLA-4 and to PD-L1 (only in TE). Furthermore, CTLA-4 correlated very well to PD-1 in TE and PD-1 TS separately, and also as one compartment (TE + TS). We did not find any correlation between miR-424-3p to subset of T cells, however, CTLA-4 were correlated to CD3+ T cells and CD4+ T cell. Some of these findings are in correlation with others^5–8,12^.

To date, there are conflicting explanation for the suboptimal responses to check-point inhibitors in PC. Studies have suggested that the high immunogenicity of PC is one reason, because PC is a slow-growing disease allowing time for a clinically relevant response.

Here, we argue for the importance of miR-424-3p expression alone, and in close correlation with CTLA-4 as negative factors for worse outcome in PC. In this study we also had the advantages of investigating TE, TS and TE + TS separately which might can be of interest to better understand the reciprocal interplay between TE and TME and their interactions with the ICPs.

In conclusion, here we demonstrate that low expression of miR-424-3p is associated with reduced clinical failure-free survival and worse outcome in PC. Furthermore, we found a significant correlation between miR-424-3p and CTLA-4. Our findings indicate that the miR-424-3p/CTLA-4 and miR-424-3p/PD-1 pathways are important in PC and that these interactions may have potential as a therapeutic target.
